# Clinical and radiographic outcomes of supracondylar humerus fractures treated surgically by pediatric and non-pediatric orthopedic surgeons

**DOI:** 10.1007/s11832-015-0642-3

**Published:** 2015-02-21

**Authors:** Seth D. Dodds, Monique A. Grey, Daniel D. Bohl, Eamonn M. Mahoney, Peter A. DeLuca

**Affiliations:** Department of Orthopaedics and Rehabilitation, Yale School of Medicine, 800 Howard Ave., New Haven, CT 06519 USA

**Keywords:** Supracondylar humerus fracture

## Abstract

**Purpose:**

This study compares clinical and radiographic outcomes of operatively managed pediatric supracondylar humerus fractures between patients treated by pediatric orthopedists (POs) and patients treated by non-pediatric orthopedists (NPOs).

**Patients and methods:**

A retrospective cohort study of pediatric patients with surgically managed supracondylar humerus fractures was conducted. For clinical outcomes analyses, 3 months of clinical follow-up were required, resulting in a sample size of 90 patients (33 treated by NPOs, 57 by POs). For radiographic outcomes analyses, 3 months of both clinical and radiographic follow-up were required, resulting in a sample size of 57 patients (23 treated by NPOs, 34 by POs).

**Results:**

The rate of inadequate fracture fixation was higher for patients treated by NPOs (43.5 %) than for patients treated by POs (14.7 %; *p* = 0.030), but rates of clinical complications, malreduction, and postoperative loss of reduction did not differ. Treatment with open reduction was more common for patients treated by NPOs (33.3 %) than for patients treated by POs (3.5 %; *p* < 0.001). Total operating room time was longer for patients treated by NPOs (110.9 min) than for patients treated by POs (82.9 min; *p* < 0.001).

**Conclusions:**

While patients treated by POs differed from patients treated by NPOs with respect to several intermediate outcomes, including having a lower rate of open reduction and a lower rate of inadequate fracture fixation, there were no differences between POs and NPOs in the rates of the more meaningful and definitive outcomes, including clinical complications, malreduction, and postoperative loss of reduction.

## Introduction

In the United States, there has been a trend towards treatment of pediatric supracondylar humerus fractures by pediatric orthopedists (POs) rather than non-pediatric orthopedists (NPOs) [1]. This is in spite of the fact that there is a well-documented shortage of POs, with more senior POs retiring than new recruits coming out of fellowship [2, 3]. Moreover, in some geographic areas, there exists a particular dearth of POs, leaving only NPOs to treat these injuries. Research in other areas of pediatric orthopedics has identified advantages of treatment by POs [4], and the outcomes of the treatment of supracondylar humerus fractures have been extensively studied with regard to closed vs. open management [5–9], immediate vs. delayed treatment [10–14], and crossed vs. lateral pin fixation [15–17].

Several studies have examined how surgeon experience impacts the outcomes of supracondylar humerus fractures. One group showed that, among fellows, non-ideal reductions increased notably at case 7, correlating with increased fellow independence in the operating room, with reversal of the trend at case 15 [18]. Another group found that while there was no poor outcome among 17 cases in which there was direct involvement of the consultant in primary management, of the 54 cases in which primary management was carried out independently by trainees without any consultant supervision, nine patients (17 %) developed complications or needed reoperations [19]. Finally, a third group found that among patients with severe fractures, patients were more likely to be treated by open reduction if treated by POs than if treated by NPOs; however, they found no other significant differences between patients treated by POs and patients treated by NPOs [20].

As part of an effort to improve the quality of surgical care, the orthopedic community should regularly ask itself for which procedures sub-specialization may improve outcomes. Answers to such questions can help to guide case allocation, practice hiring, call schedules, and even the decisions of graduating trainees and young graduates involving which skill sets to pursue. It also can contribute in general to our understanding of the impact of sub-specialization on the field.

In this context, among pediatric patients treated operatively for supracondylar humerus fractures, the study that follows compares outcomes between patients treated by NPOs and patients treated by POs. Our main hypotheses were that patients treated by POs and patients treated by NPOs would have different rates of clinical complications, malreduction, and postoperative loss of reduction.

## Materials and methods

A retrospective cohort study was conducted comparing patients treated by NPOs with patients treated by POs. Patients were initially identified through a current procedural terminology code search of our institution’s billing database to identify patients who had supracondylar humerus fractures operatively treated between January 1, 1994 and March 1, 2007. Among these identified cases, inclusion criteria were (1) skeletal immaturity determined by open physes and age less than 13 years, (2) documentation of at least one preoperative and at least one postoperative neurovascular examination, (3) documentation of range of motion at follow-up, (4) adequate immediate postoperative and follow-up radiographs, (5) fractures lacking a separate condylar component, (6) fractures lacking intra-articular or diaphyseal involvement, and (7) at least 3 months of clinical follow-up. To be included in radiographic outcomes analyses, patients must have also had at least 3 months of radiographic follow-up. A PO was defined as an orthopedic surgeon who had completed a fellowship in pediatric orthopedic surgery. A NPO was defined as an orthopedic surgeon who had not completed a fellowship in pediatric orthopedic surgery.

Emergency room records, inpatient charts, operative reports, and outpatient charts were examined and relevant data were extracted. The actual time of injury was rarely available; for this reason, the triage time was used as “time zero” to calculate the time elapsed between injury and surgery. Some patients had been scheduled electively for surgery as outpatients (and therefore bypassed the emergency room); in these cases, the time of their injury (and, when not recorded, the time of their office visit) was used to calculate time elapsed between injury and surgery. The range of motion at the time of final follow-up was noted and categorized as functional or nonfunctional range of motion. As defined by Morrey et al. [21], in order to be categorized as having functional range of motion, patients must have been able to attain elbow flexion through the range of 30–130° and pronation/supination through the range of 50–50°.

Preoperative and postoperative radiographs were also examined. Preoperative radiographs were used to classify fracture type according to the Wilkins modification of the Gartland classification system. If no preoperative radiograph was available, the fracture type as documented by the surgeon in the operative report was used. Fracture fixation technique was graded as adequate or inadequate according to the recommendations of Skaggs et al. [22, 23]. Fixation was considered inadequate if any of the following were noted on the postoperative radiographs: (1) pins crossing at the fracture site, (2) a pin without bicortical purchase, or (3) pins with minimal separation between their entrance sites. A fracture was considered to be malreduced if any of the following criteria were met: (1) the anterior humeral line passed either anterior or posterior to the capitellum, (2) the distal fracture fragment was malrotated, or (3) the Baumann angle was outside the range of normal values (64–82°) [24, 25]. Loss of fracture reduction was determined by comparing the immediate postoperative and follow-up radiographs. A change in position of the anterior humeral line from transection of the capitellum in its middle, anterior, or posterior one-third to a position anterior or posterior to the capitellum (i.e., missing it altogether) was considered a loss of reduction. A change in the Baumann angle from within the normal range to outside of the normal range was considered a loss of reduction if the change in the angle was more than 6°. If a change in the Baumann angle of less than 6° resulted in an abnormal value, the fracture was re-classified as initially malreduced. The rationale for this distinction is rooted in the observation that the Baumann angle varies 6° for every 10° of humeral rotation on the AP radiograph [25]. If, for example, a fracture had a marginally normal Baumann angle of 82° immediately after surgery and an abnormal Baumann angle of 86° at the time of follow-up, this small 4° change is not likely to represent a true loss of reduction, but rather an initial malreduction that was not detected because of variation in radiographic technique.

Categorical variables were analyzed using the Fisher exact test and continuous variables with equal variance were analyzed using the Student *t* test. Continuous variables with unequal variance were compared using the Mann–Whitney U test. A *p*-value <0.05 was considered to be statistically significant. All tests were two-tailed.

## Results

A total of 143 patients met initial inclusion criteria (criteria numbered 1 through 6 listed in “Methods”). Of these, 90 (62.9 %) had clinical follow-up of at least 3 months (criterion numbered 7). These 90 patients represent the “full cohort for clinical analyses,” as depicted in Table 1. Of these patients, 33 were treated by NPOs and 57 were treated by POs. Among these patients, there were no differences in baseline characteristics between patients treated by NPOs and POs, with one exception: the proportion of patients who were electively scheduled outpatients was lower for patients treated by NPOs (0.0 %) than for patients treated by POs (14.0 %; *p* = 0.025; Table 1).

**Table 1:** Study population

	Full cohort for clinical analyses (minimum of 3 months of clinical follow-up; *N* = 90)			Restricted cohort for radiographic analyses (minimums of 3 months of clinical and radiographic follow-up; *N* = 57)		
	NPO (*N* = 33)	PO (*N* = 57)	*p* value	NPO (*N* = 23)	PO (*N* = 34)	*p* value
Average age (years)	5.9 ± 1.9 (3–10)	5.7 ± 2.0 (1–10)	0.678	5.8 ± 1.6 (4–9)	5.6 ± 2.2 (1–10)	0.800
Sex			0.510			0.018
Male	17 (51.5 %)	24 (42.1 %)		15 (65.2 %)	11 (32.4 %)	
Female	16 (48.5 %)	33 (57.9 %)		8 (34.8 %)	23 (67.7 %)	
Transfer from outside institution			0.435			0.724
No	24 (72.7 %)	46 (80.7 %)		20 (87.0 %)	27 (79.4 %)	
Yes	9 (27.3 %)	11 (19.3 %)		3 (13.0 %)	7 (20.6 %)	
Electively-scheduled outpatient			0.025			0.071
No	33 (100.0 %)	49 (86.0 %)		23 (100.0 %)	28 (82.4 %)	
Yes	0 (0.0 %)	8 (14.0 %)		0 (0.0 %)	6 (17.7 %)	
Fracture type			0.073			0.145
Type II	12 (36.4 %)	10 (17.5 %)		9 (39.1 %)	7 (20.6 %)	
Type III	21 (63.6 %)	47 (82.5 %)		14 (60.9 %)	27 (79.4 %)	
Ipsilateral upper extremity fracture			0.409			0.265
No	32 (97.0 %)	52 (91.2 %)		23 (100.0 %)	31 (91.2 %)	
Yes	1 (3.0 %)	5 (8.8 %)		0 (0.0 %)	3 (8.8 %)	
Both-bones forearm fracture	1	2		0	1	
Distal radius fracture	0	3		0	2	
Preoperative nerve injury			0.524			0.689
No	30 (90.9 %)	48 (84.2 %)		21 (91.3 %)	29 (85.3 %)	
Yes	3 (9.1 %)	9 (15.8 %)		2 (8.7 %)	5 (14.7 %)	
Anterior interosseous nerve	1	5		1	3	
Median nerve	1	0		1	0	
Radial nerve	0	2		0	2	
Ulnar nerve	1	2		0	0	
Pink, pulseless hand			0.409			0.265
No	32 (97.0 %)	52 (91.2 %)		23 (100.0 %)	31 (91.2 %)	
Yes	1 (3.0 %)	5 (8.8 %)		0 (0.0 %)	3 (8.8 %)	
Open fracture			1.000			1.000
No	33 (100.0 %)	57 (100.0 %)		23 (100.0 %)	34 (100.0 %)	
Yes	0 (0.0 %)	0 (0 %)		0 (0.0 %)	0 (0 %)	
Closed reduction in emergency room			0.082			0.510
No	33 (100.0 %)	51 (89.5 %)		23 (100.0 %)	32 (94.1 %)	
Yes	0 (0.0 %)	6 (10.5 %)		0 (0.0 %)	2 (5.9 %)	
Duration of clinical follow-up (months)	10.2 ± 8.3 (3–26)	9.3 ± 7.3 (3–32)	0.562	N/A	N/A	N/A
Duration of radiographic follow-up (months)	N/A	N/A	N/A	13.3 ± 13.2 (3–48)	12.7 ± 12.4 (3–50)	0.879

Continuous variables have values given as mean ± standard deviation (range) and are compared using *t* tests. Categorical variables are compared using the Fisher exact test

*NPO* non-pediatric orthopedist, *PO* pediatric orthopedist, *N/A* not applicable

A total of 57 patients had both clinical follow-up of at least 3 months and radiographic follow-up of at least 3 months. These patients represent the “restricted cohort for radiographic analyses,” as depicted in Table 1. Of these patients, 23 were treated by NPOs and 34 were treated by POs. Among these patients, there were no differences in baseline characteristics between patients treated by NPOs and POs, with one exception: the proportion of patients who were female was lower for patients treated by NPOs (34.8 %) than for patients treated by POs (67.7 %; *p* = 0.018; Table 1).

Surgical management strategies and hospital courses are compared between patients treated by NPOs and patients treated by POs in Table 2. Treatment with open reduction was more common for patients treated by NPOs (33.3 %) than for patients treated by POs (3.5 %; *p* < 0.001). Total operating room time was longer for patients treated by NPOs (110.9 min) than for patients treated by POs (82.9 min; *p* < 0.001).

**Table 2:** Surgical and hospital data (*N* = 90)

	NPO (*N* = 33)	PO (*N* = 57)	*p* value
Time elapsed to surgery (h)	6.0 ± 4.5 (1.2–24.0)	18.7 ± 42.0 (0.8–288.0)	0.060
Open reduction			<0.001
No	22 (66.7 %)	55 (96.5 %)	
Yes	11 (33.3 %)	2 (3.5 %)	
Pin configuration			0.337
Crossed (medial and lateral)	26 (78.8 %)	39 (68.4 %)	
Lateral	7 (21.2 %)	18 (31.6 %)	
Convergent	0	1	
Divergent	4	10	
Parallel	3	5	
Total operating room time (min)	110.9 ± 40.0 (60–215)	82.9 ± 24.6 (47–190)	<0.001
Length of hospital stay (days)	1.3 ± 0.5 (1–3)	1.1 ± 0.7 (0–3)	0.074

Continuous variables have values given as mean ± standard deviation (range) and are compared using Mann–Whitney *U* tests (time elapsed to surgery) or *t* tests (total operating room time and length of hospital stay). Categorical variables are compared using the Fisher exact test

*NPO* Non-pediatric orthopedist, *PO* Pediatric orthopedist

Rates of complications are compared between patients treated by NPOs and patients treated by POs in Table 3. Patients treated by NPOs and patients treated by POs did not have different rates of individual complications and did not have different rates of presence of any complications. Qualities of iatrogenic nerve injuries are detailed in Table 4.

**Table 3:** Complications (*N* = 90)

	NPO (*N* = 33)	PO (*N* = 57)	*p* value
Iatrogenic nerve injury^a^			0.740
No	30 (90.9 %)	50 (87.7 %)	
Yes	3 (9.1 %)	7 (12.3 %)	
Infection^b^			1.000
No	33 (100.0 %)	56 (98.3 %)	
Yes	0 (0.0 %)	1 (1.8 %)	
Reoperation^c^			1.000
No	32 (96.5 %)	54 (94.7 %)	
Yes	1 (3.5 %)	3 (5.3 %)	
Refracture			1.000
No	33 (100.0 %)	57 (100.0 %)	
Yes	0 (0.0 %)	0 (0.0 %)	
Deformity^d^			0.367
No	32 (97.0 %)	57 (100.0 %)	
Yes	1 (3.0 %)	0 (0.0 %)	
Compartment syndrome			1.000
No	33 (100.0 %)	57 (100.0 %)	
Yes	0 (0.0 %)	0 (0.0 %)	
Any clinical complications			1.000
No	29 (87.9 %)	48 (84.2 %)	
Yes	4 (12.1 %)	9 (15.8 %)	

Variables are compared using the Fisher exact test. “Any clinical complications” is a composite outcome that is “Yes” for patients who had at least one complication and “No” for patients who had no complications

*NPO* non-pediatric orthopedist, *PO* pediatric orthopedist

^a^*Iatrogenic nerve injury* Qualities of iatrogenic nerve injuries are detailed in Table 4

^b^*Infection* The single patient with infection had a deep infection with concomitant osteomyelitis that was diagnosed 6 weeks postoperatively and was treated successfully with incision, debridement, and intravenous antibiotics. The patient went on to uneventful healing with functional range of motion at the time of final follow-up

^c^*Reoperation* The reoperation in the patient treated by an NPO was an anterior elbow release for contracture. Reasons for each of the three reoperations in patients treated by POs were (1) manipulation under anesthesia for elbow stiffness, (2) incision and debridement for deep infection (same patient as that described as having had infection as a complication), and (3) medial pin removal for ulnar dysesthesias (same patient as one of the patients described as having had iatrogenic nerve injury as a complication)

^d^*Deformity* The single patient who had a deformity had a noticeable cubitus valgus deformity. Of note, the fracture was initially inadequately reduced. At the time of final follow-up, the patient was asymptomatic and had no functional limitations

**Table 4:** Qualities of iatrogenic nerve injuries (*N* = 90)

	NPO (*N* = 33)	PO (*N* = 57)
Nerve injured^1^		
Ulnar	2	5
Radial	0	2
Median	1	1
Pin configuration		
Crossed	2	7
Lateral	1	0

*NPO* non-pediatric orthoaedist, *PO* pediatric orthopedist

^1^One patient who was treated by a PO had combined nerve symptoms

Rates of nonfunctional range of motion are compared between patients treated by NPOs and patients treated by POs in Table 5. Rates of nonfunctional range of motion did not differ between patients treated by NPOs and patients treated by POs.

**Table 5:** Range of motion (*N* = 90)

	NPO (*N* = 33)	PO (*N* = 57)	*p* value
Range of motion			0.255
Functional	29 (87.9 %)	54 (94.7 %)	
Nonfunctional	4 (12.1 %)	3 (5.3 %)	

Variables are compared using the Fisher exact test

*NPO* non-pediatric orthopedist, *PO* pediatric orthopedist

**Fig. 1:**
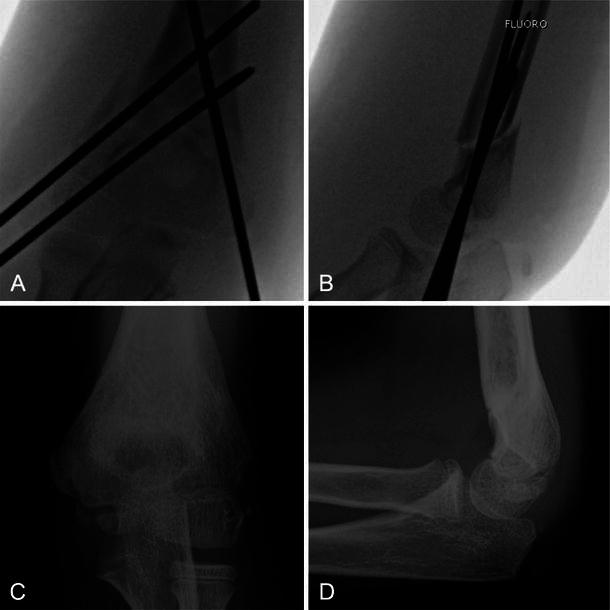
Adequate fracture fixation and appropriate reduction. **a** AP with pins. **b** Lateral with pins. **c** AP after healing. **d** Lateral after healing

**Fig. 2:**
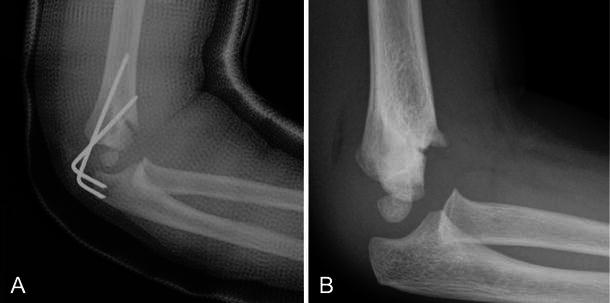
Inadequate fracture fixation with pins having minimal separation between their entrance sites and malreduction with the anterior humeral line anterior to the capitellum. **a** Lateral with pins. **b** Lateral after healing

Radiographic outcomes are compared between patients treated by NPOs and patients treated by POs in Table 6. Of note, unlike for all previously listed results, the following analysis was conducted among the restricted cohort instead of the full cohort. The restricted cohort required both a minimum of 3 months of radiographic follow-up and a minimum of 3 months of clinical follow-up. There were no differences in radiographic outcomes between patients treated by NPOs and patients treated by POs, with one exception: patients treated by NPOs were more likely to have inadequate fracture fixation (43.5 %) than patients treated by POs (14.7 %; *p* = 0.030). Examples of adequate and inadequate fracture fixation are shown in Figs. 1 and 2, respectively. The case with adequate fixation (Fig. 1) was appropriately reduced and had no postoperative loss of reduction. The case with inadequate fixation (Fig. 2) had pins with minimal separation between their entrance sites. It was also malreduced with the anterior humeral line anterior to the capitellum.

**Table 6:** Radiographic outcomes (*N* = 57)

	NPO (*N* = 23)	PO (*N* = 34)	*p* value
Inadequate fracture fixation			0.030
No	13 (56.5 %)	29 (85.3 %)	
Yes	10 (43.5 %)	5 (14.7 %)	
Pins crossing at the fracture site	3	0	
Pin without bicortical purchase	5	2	
Pins with minimal separation between their entrance sites	0	3	
Multiple factors	2	0	
Malreduction			0.124
No	20 (87.0 %)	23 (67.7 %)	
Yes	3 (13.0 %)	11 (32.4)	
Abnormal Baumann angle	1	2	
Anterior humeral line anterior or posterior to capitellum	0	4	
Malrotation	2	5	
Postoperative loss of reduction			0.058
No	19 (82.6 %)	33 (97.1 %)	
Yes	4 (17.4 %)	1 (2.9 %)	
Abnormal Baumann angle	2	1	
Anterior humeral line anterior or posterior to capitellum	1	0	
Malrotation	1	0	
Postoperative Baumann angle (°)	73.4 ± 5.0	74.1 ± 5.9	0.752
Follow-up Baumann angle (°)	71.9 ± 5.5	72.6 ± 6.7	0.645
Change in Baumann angle (°)	6.3 ± 4.3	5.1 ± 3.8	0.306

Continuous variables have values given as mean ± standard deviation and are compared using *t* tests. Categorical variables are compared using the Fisher exact test

*NPO* non-pediatric orthopedist, *PO* pediatric orthopedist

## Discussion

There has been a trend towards treatment of pediatric supracondylar humerus fractures by POs rather than NPOs [1], despite a shortage of POs in the workforce [2, 3]. The quality of surgical care provided to orthopedic patients might be improved by identification of any advantages associated with sub-specialist performance of common orthopedic procedures. Such improvements might manifest through optimization of case allocation, surgeon hiring, or call schedules. Supracondylar humerus fractures are the most common operative fractures in children [26]; hence, any potential advantages of treatment by POs could have important implications for patient care. The purpose of this study was to test for the presence of such advantages in a cohort of surgically managed patients. In summary, this study did not demonstrate differences between patients treated by POs and patients treated by NPOs in the rates of the most meaningful clinical and radiographic outcomes (our main hypotheses), including clinical complications, malreduction, and postoperative loss of reduction. There were significantly higher rates of open reduction and inadequate fracture fixation in patients treated by NPOs than in patients treated by POs, but the differences in rates of these intermediate outcomes appear to have had minimal clinical consequence.

Patients treated by NPOs and POs had some differences in terms of surgical management and hospital course. Specifically, patients treated by NPOs were more likely than those treated by POs to undergo open reductions, typically for failure of closed reduction. This is likely related to the greater degree of experience that POs have with these cases. Most studies that have reviewed the results of supracondylar humerus fractures treated with open reduction have reported satisfactory clinical and radiographic outcomes [5–9], so this difference may have minimal clinical consequence in and of itself. The more than 20 min longer that patients treated by NPOs spent in the operating room is similarly most likely due to the difference in experience, and the clinical impact of the difference in this intermediate outcome may be minimal.

The present study does not demonstrate differences in the rates of clinical complications between patients treated by NPOs and those treated by POs. This result is in line with the only other study that has investigated this question, Farley et al. [20]; however, those authors only compared a composite rate of “any complications”—they did not compare the specific rates of individual complications as compared here. The present study nearly doubles the total number of patients that have been studied with respect to this question.

Similarly, the present study does not demonstrate a difference in the rate of nonfunctional range of motion between patients treated by NPOs and those treated by POs. This is an additional important piece of evidence to suggest that outcomes may be similar after treatment by NPOs and POs. This outcome was not reported on by Farley et al. [20] and so is unique in the literature.

Finally, the present study does not demonstrate differences in the majority of radiographic outcomes between patients treated by NPOs and those treated by POs. Most importantly, no significant differences were found in the rates of fracture malreduction or postoperative loss of reduction between the two groups. This is a third piece of evidence to suggest that outcomes may be similar after treatment by NPOs and POs. Although this study found higher rates of inadequate fracture fixation (fixation not adhering to the recommendations of Skaggs et al. [22, 23]) in patients treated by NPOs, the implications of this are likely minimal in the setting of similar rates of malreduction and postoperative loss of reduction, outcomes with better defined clinical implications. Given the very high rates of inadequate fracture fixation based on these criteria (43.5 % for patients treated by NPOs and 14.7 % for patients treated by POs), and given the absence of observed clinical consequences, these criteria may have limited clinical relevance.

The study does have limitations. First, as it was conducted at a single institution, the surgeons who contributed patients may not have been representative of other NPOs and POs. Second, clinical and radiographic follow-up were limited to 3 months; however, the duration of follow-up was similar between patients treated by NPOs and patients treated by POs, so we have no reason to believe that the cases with limited follow-up would have necessarily biased our results towards one type of practitioner or the other. Third, while the populations of patients treated by NPOs and POs were similar in terms of most measured baseline characteristics (Table 1), it is possible that there were differences in baseline characteristics that were not measured.

Despite showing some differences in intermediate outcomes, including rates of open reduction and inadequate fracture fixation, this study did not demonstrate differences in the rates of most clinical and radiographic outcomes, including clinical complications, malreduction, and postoperative loss of reduction, between patients treated by NPOs and patients treated by POs. This is reassuring in the setting of the reported shortage of POs because, particularly in under-populated areas, access to POs may not always be possible.
